# Enhancing Extractable Quantum Entropy in Vacuum-Based Quantum Random Number Generator

**DOI:** 10.3390/e20110819

**Published:** 2018-10-24

**Authors:** Xiaomin Guo, Ripeng Liu, Pu Li, Chen Cheng, Mingchuan Wu, Yanqiang Guo

**Affiliations:** 1 Key Laboratory of Advanced Transducers and Intelligent Control System, Ministry of Education, Taiyuan 030024, China; 2 College of Physics and Optoelectronics, Taiyuan University of Technology, Taiyuan 030024, China

**Keywords:** quantum random number, vacuum state, maximization of quantum conditional min-entropy

## Abstract

Information-theoretically provable unique true random numbers, which cannot be correlated or controlled by an attacker, can be generated based on quantum measurement of vacuum state and universal-hashing randomness extraction. Quantum entropy in the measurements decides the quality and security of the random number generator (RNG). At the same time, it directly determines the extraction ratio of true randomness from the raw data, in other words, it obviously affects quantum random bits generating rate. In this work, we commit to enhancing quantum entropy content in the vacuum noise based quantum RNG. We have taken into account main factors in this proposal to establish the theoretical model of quantum entropy content, including the effects of classical noise, the optimum dynamical analog-digital convertor (ADC) range, the local gain and the electronic gain of the homodyne system. We demonstrate that by amplifying the vacuum quantum noise, abundant quantum entropy is extractable in the step of post-processing even classical noise excursion, which may be deliberately induced by an eavesdropper, is large. Based on the discussion and the fact that the bandwidth of quantum vacuum noise is infinite, we propose large dynamical range and moderate TIA gain to pursue higher local oscillator (LO) amplification of vacuum quadrature and broader detection bandwidth in homodyne system. High true randomness extraction ratio together with high sampling rate is attainable. Experimentally, an extraction ratio of true randomness of 85.3% is achieved by finite enhancement of the laser power of the LO when classical noise excursions of the raw data is obvious.

## 1. Introduction

Randomness is one vital ingredient in modern information science, in the regime of both classical and quantum [1,2], since encryption is founded upon the trust in random numbers [3,4,5]. The demand for true and unique randomness in these applications has triggered various proposals for producing random numbers based on the measurements of quantum observables, which offer the verifiability and ultimate in randomness. In the past two decades, there has been tremendous development for various types of quantum RNG [6,7,8,9,10,11,12,13,14,15]. Among these proposals, random number generation based on homodyne measurement of quantum vacuum state is especially appealing in practice since highly efficient photodiodes working at room temperature can be applied [11]. Vacuum state is a pure quantum state with the lowest energy and independent of any external physical quantities. It cannot be correlated or controlled by an attacker, therefore unique random numbers can be yielded by measuring the quadrature amplitude of the vacuum state [16,17]. All the components in this scheme, including laser source, beam splitter and photo detectors have been integrated on a single chip recently [18]. Meanwhile, bit conversion and post-processing are easy to be implemented in virtual “hardware” inside the field-programmable gate array (FPGA). Chip-size integration of the QRNG is expectable. Several dedicated researches have been developed to enhance the generation rate of random bits in this proposal, such as schemes based on optimization of the digitization algorithm [19], implementation of fast randomness extraction in the post-processing [20], application of squeezing vacuum state to increase entropy in raw data [21] and optimization of ADC parameters to improve the quantum entropy in the raw data [22]. In this paper, considering the effects of the classical noise, we discuss the role of homodyne gain in enhancing quantum entropy in the vacuum-based quantum RNG working in the optimum dynamical ADC range scenario. Conditional min-entropy is applied to critically assess the quantum entropy in the quantum RNG. It is the key input parameter of randomness extractor and determines the extraction ratio of true randomness from the raw random sequence, thereby affects the generation speed of quantum RNG significantly.

## 2. Quantum Entropy Evaluation and Enhancing in Vacuum-Based Quantum RNG

Entropy is defined relative to one’s knowledge of an experiment’s output prior to observation. The larger the amount of the entropy, the greater the uncertainty in predicting the value of an observation. Among types of entropy, min-entropy is a very conservative measure. In cryptography, the unpredictability of secret values is essential. The min-entropy measure the probability that a secret is guessed correctly in the first trial. For mathematically determining min-entropy of a secret, the first thing is to precisely identify the distribution that the secret was generated from [23].

Quadrature fluctuation of optical quantum vacuum state, the nature initial state of optical field at room temperature, is the noise source for the random bits generation in this scheme. According to Born’ s rule, the measurement outcome of a pure quantum state can be intrinsically random. A single measurement of the quadrature of the vacuum state is completely random and multiple repeated measurements satisfy the Gaussian distribution statistically, so we can extract random bits from the measurement results. Based on homodyne measurement, the microscopic fluctuations of quadrature of the vacuum state are detected, amplified and transferred into an electric signal

(1) $$V_{\mathrm{vac}}\propto \langle i_{-}{}^{2}\rangle -{\langle i_{-}\rangle }^{2}\propto 4\alpha^{2}[\delta X{\left(t\right)}^{2}\cos{\left(\theta \right)}^{2}+\delta Y{\left(t\right)}^{2}\sin{\left(\theta \right)}^{2}]$$

$i_{-}$ is the difference current from the two detectors. Measured quantum quadrature of vacuum state in any local phase is amplified by the factor $\alpha^{2}{=g}_{TIA}\alpha_{L}{}^{2}$, which includes the amplification effects from LO gain and electronics gain in the system [24]. Without regard to classical noise, the electric signals (voltage or current) obey a Gaussian distribution:

(2) $$P(V_{\mathrm{vac}})=\frac{1}{\sqrt{\pi }\alpha }\exp(-\frac{V_{\mathrm{vac}}{}^{2}}{\alpha^{2}}).$$

The coefficient α has to be calibrated to rescale histogram of the associated marginal distribution in optical homodyne tomography (OHT) [25]. In this scheme of quantum random numbers generation, α is associated with the quantum entropy contained in the measured data and it is the critical parameter for digitization of the measured analogue signal. 

When classical noise is taken into account, such as electronic noise and local noise resulted from imperfect balancing in balanced homodyne detection (BHD), the observed probability distribution of the electric signal is in the form of a convolution of the scaled vacuum state marginal distribution and the classical noise histogram

(3) $$P_{\mathrm{obs}}(V)=\frac{1}{\alpha }\int P(\frac{V^{\prime }}{\alpha })P_{\mathrm{cl}}(V-V^{\prime })dV^{\prime }.$$

without loss of generality, the broadband electric noise and the LO noise distribution can be assumed to be Gaussian:

(4) $$P_{\mathrm{cl}}(V_{\mathrm{cl}})=\frac{1}{\sqrt{\pi B}}\exp(-\frac{V_{\mathrm{cl}}{}^{2}}{B}).$$

The vacuum noise and the classical noise as two variables with normal distribution, are independent with each other, thus their sum is also normally distributed with a total variance equal to the sum of the two variances.

According to Equations (2)–(4), the homodyne measurement of the vacuum state yields a signal distribution as follows

(5) $$P_{\mathrm{obs}}(V)=\frac{1}{\sqrt{\pi }\sqrt{\alpha^{2}+B}}\exp(-\frac{V^{2}}{\alpha^{2}+B}),$$

with the measurement variance of

(6) $$\sigma_{\mathrm{obs}}^{2}=\sigma_{\mathrm{quan}}^{2}+\sigma_{\mathrm{cl}}^{2}=\left(\alpha^{2}+B\right)/2,$$

where factor 2 is added to renormalize the distribution. Then the quantum and classical noise ratio (*QCNR*) in the homodyne measurement system is defined as

(7) $$QCNR=10\mathrm{Lg}(\sigma_{\mathrm{quan}}^{2}/\sigma_{\mathrm{cl}}^{2}).$$

The *QCNR* related to the signal-to-noise ratio of homodyne detection, is defined as the ratio between the mean square noise of the measured vacuum state and the electronic noise, that is, the quantity

(8) $$S=\left(\alpha^{2}+B\right)/B=\sigma_{o\mathrm{bs}}^{2}/\sigma_{\mathrm{cl}}^{2},$$

or the clearance between the shot noise power spectrum and electronic noise power in dB units, ${10\mathrm{Log}}_{10}(S)\;\mathrm{dB}$, reads on spectrum analyzer. In other words, when the homodyne detection system works in linear region, the *QCNR* of the raw data can be indicated from the clearance shown by spectrum analyzer.

**Figure 1:**
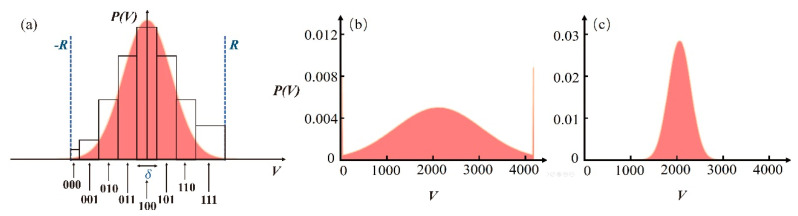
(**a**) Model of 3-bit analog-digital converter (ADC); (**b**) Numerical simulations of acquisition conditions for a Gaussian signal when dynamical ADC range is chosen too small; (**c**) too big.

In our proposal, as a continuous-variable, the measurement output consisting of scaled quadrature of the vacuum state and the classical noise is discretized by an *n*-bit ADC with a dynamical range [−R+δ/2,R−3δ/2]. The sampled signals are binned over $2^{n}$ bins with width of $\delta =R/2^{n-1}$ and are assigned a corresponding bit combination with length of *n*. 3-bit ADC binning is shown in Figure 1a as an example.

In order to design an entropy source that provides an adequate amount of entropy per output bit string, the developer must be able to accurately estimate the amount of entropy that can be provided by sampling its noise source. The behavior of the other components included in the entropy must also be known clearly since the behavior of the other components may affect the assessment of the entropy. In our system, the randomness or the entropy in the measurements could derive from multiple factors, such as the quantum fluctuation, classical influences on it and even malicious attack from the third part [5]. Especially and strictly, quantum conditional min-entropy is used to evaluate the maximal amount of randomness extractable from the total entropy of the system [26]. Firstly, the min-entropy for the Gaussian distribution is defined as

(9) $$H_{\min}(X)=-{\mathrm{Log}}_{2}{(\max}_{V\in {\left\{0,1\right\}}^{n}}\mathrm{Prob}[X=V]).$$

In this scheme, the min-entropy of the probability distribution of quadrature measurements can be accurately predicted from the probability density function of the quantum signal. The maximum probability in (9) can be acquired based on the probability distribution discretized by the bins

(10) $$P_{\mathrm{bin}}(V_{i})=\{\begin{array}{c} \int_{-\infty }^{-R+\delta /2}P_{\mathrm{obs}}(V)dV,i=i_{L},\\ \int_{V_{i}-\delta /2}^{V_{i}+\delta /2}P_{\mathrm{obs}}(V)dV,i_{L}<i<i_{M},\\ \int_{R-3\delta /2}^{+\infty }P_{\mathrm{obs}}(V)dV,i=i_{M}. \end{array}$$

Each bin is labelled by an integer $i\in \{i_{L},...,i_{M}\}$, with $i_{L}=-2^{n-1}$, the least significant bits (LSB) bin, $i_{M}=2^{n-1}-1$, the most significant bit (MSB) bin and $V_{i}=i\times \delta$.

Secondly, some restrictions must be taken into account in analog-digital conversion process. Those samples go off-scale, that is, points in saturation will be recorded as extrema values as depicted in Figure 1b. So, underestimating the range will induce too many blocks of zeros and ones. Conversely, overestimating the signal range will lead to undue unused bins (Figure 1c). In either situation, some bit combinations are too frequent to be considered random. It is necessary to adjust the amplitude of the analogue signal and the ADC dynamical range in order to employ the full *n*-bit sampling properly whenever possible.

Further, considering the influence of classical noise on the measurement outcome, ADC dynamical range should be optimized over the classical noise shifted quantum signal probability distribution. In application scenario, inevitable classical noise excursion in the measurement system will result in nonzero mean in the measured signal probability distribution. On the other hand, eavesdropper may induce a deliberate offset over the sampling period. In a word, a noticeable classical noise excursion, Δ, need to be considered in the optimization of the sampling dynamical range.

Taking into account all these factors offered above, we rewrite the discretized probability distribution as,

(11) $$P_{\mathrm{bin}}(V_{i}|V_{cl})=\{\begin{array}{c} \int_{-\infty }^{-R+\delta /2-\Delta }P(V_{i}|V_{cl})dV,i=i_{L},\\ \int_{V_{i}-\delta /2-\Delta }^{V_{i}+\delta /2-\Delta }P(V_{i}|V_{cl})dV,i_{L}<i<i_{M},\\ \int_{R-3\delta /2-\Delta }^{+\infty }P(V_{i}|V_{cl})dV,i=i_{M}. \end{array}$$

where,

(12) $$P(V_{i}|V_{cl})=\frac{1}{\sqrt{\pi }\alpha }\exp(-\frac{{\left(V-V_{cl}\right)}^{2}}{\alpha^{2}})\;$$

is the probability density distribution of the quantum signal given full knowledge of the classical noise $V_{\mathrm{cl}}$, where $V_{\mathrm{cl}}\in [V_{\mathrm{cl},\min},V_{cl,\max}]$ with an excursion of Δ. Finally, the quantum conditional min-entropy is expressed as

(13) $$\begin{array}{l} H_{\min}(V|V_{\mathrm{cl}})=-{\mathrm{Log}}_{2}[\mathrm{Max}(\frac{1}{2}\{1+\mathrm{Erf}[\frac{-2(V_{\mathrm{cl},\min}+R+\Delta )+\delta }{2\alpha }]\},\\ \mathrm{Erf}(\frac{\delta }{2\alpha }),\frac{1}{2}\{1+\mathrm{Erf}[\frac{2(V_{\mathrm{cl},\max}-R+\Delta )+3\delta }{2\alpha }]\})]. \end{array}$$

In the best-case scenario of ADC sampling range, the measurement outcome probability in the center bin is equal to the higher one of the first and the last bins. In this way, the quantum conditional min-entropy is information theoretically provably estimated and the amount of quantum-based randomness in the total noise signal is rigorously evaluates. In applications with the requirement of information security, a random sequence is demanded to be truly unpredictable and have maximum entropy [27].

At the same time, the conditional min-entropy sets the lower bound of extractable randomness from the raw measurements and quantifies the least amount of randomness possessed by each sample or $P=H_{\min}(X)/n$ bit per raw bit. Quantum randomness can be distilled from raw data by applying information theoretically provable Toeplitz-hash extractor. As discussed above, the key point is to find out the *QCNR* and derive the probability distribution of the quantum signal. The higher the *QCNR*, the more true randomness can be extracted from the raw measurement. Only when *QCNR* is high enough, both the quality and the security of the random number generator are guaranteed. Fulfilling the condition of optimal dynamical sampling range *R*, minimum-entropy of the quantum signal for growing clearance is theoretically analyzed. Proceeding from the directly measurable quantity, homodyne clearance, corresponding *QCNR* is derived from Equation (8). Then quantum noise variances are expressed as multiples of the $\sigma_{cl}$. For different clearance, probabilities of middle bin and the LSB/MSB are compared and the optimal sampling range *R* is decided based on Equation (11). Finally, based on Equation (13), the quantum conditional min-entropy in optimal sampling range scenario as a function of different classical noise excursion is analyzed.

**Figure 2:**
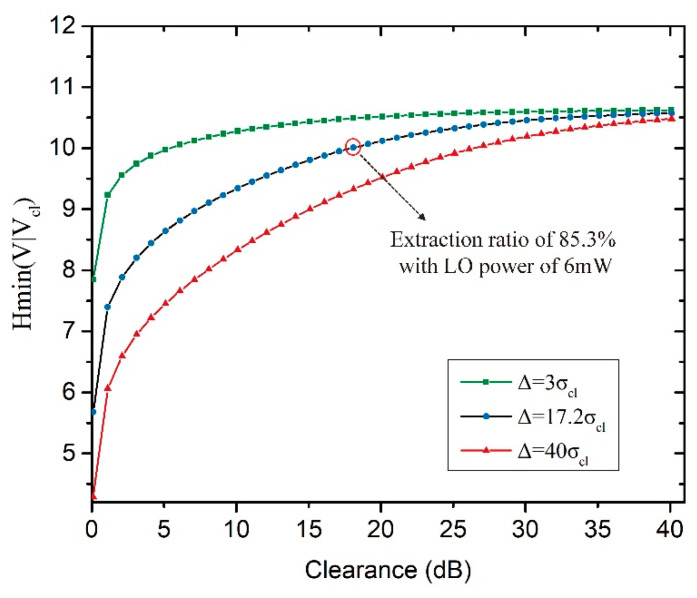
Optimized $H_{\min}\left(V|V_{\mathrm{cl}}\right)$ as a function of homodyne detection clearance among different classical excursions. The theoretical value circled in red corresponding to the highest extraction ratio of true randomness in our experiment.

The classical noise excursions in our raw data have been collected from multiple measurements, which range from almost 3 to 29 times of classical noise standard deviation $\sigma_{cl}$. In application scenario, much larger DC offset may be induced deliberately by the eavesdropper. In Figure 2, we show the quantum conditional min-entropy, $H_{\min}\left(V|V_{\mathrm{cl}}\right)$, as a function of homodyne detection clearance for three different classical noise excursions under the precondition of optimal sampling range. $\Delta =3\sigma_{cl}$ is the smallest classical noise excursion among our multiple measurements, $\Delta =40\sigma_{cl}$, a larger classical noise excursion for comparison and $\Delta =17.2\sigma_{cl}$ is the excursion in the raw data from which we extract true random numbers. As shown in Figure 2, the extractable random bits are robust against the decline of *QCNR* while the classical excursion is subtle. Whereas if classical noise excursion is evidence, one can achieve high secure randomness only when clearance is high enough.

The clearance relies on the total gain in homodyne detection system (also α in Equation (1)), including the LO amplification and the electrical gain. In quantum state measurements and reconstructions, the clearance needed between shot noise and classical noise is dependent on the amount of squeezing and entanglement one wishes to measure. Empirically, the homodyne system should satisfy the condition that the measured shot noise is 10 dB higher than the classical noise among the analysis frequency range [28,29]. High TIA gain and moderate dynamical range are required so that shot noise is the dominant spectral feature among the detection frequency range. In this scheme of quantum RNG, however, high *QCNR,* but also large detection bandwidths, are pursued, since the cut-off frequency of the homodyne detector upper bound the sampling frequency in random numbers generation process [30].

On the other hand, the classical effects, which blur the distribution and cause classical entropy in the raw bit sequence, include imperfect balancing of LO, non-unit quantum efficiency and electronic noise of the detectors [31,32,33,34]. The non-unit detector efficiency can almost completely overcome by using special fabricated diodes and the quantum efficiencies of more than 99% have been reported [35]. The detrimental electronic noise depends on numerous components in the circuit part as expressed by

(14) $$V_{\mathrm{EL},\mathrm{noise}}=R\sqrt{(4KT/R_{\mathrm{PD}}+I_{\mathrm{PD},\mathrm{dk}}^{2}+4KT/R_{r}+I_{\mathrm{TIA},c}^{2})+{\left(V_{\mathrm{TIA},v}^{}/R\right)}^{2}}$$

One term is from the photodiode (PD) and comprise of thermal noise and dark current noise of PD, both of which are usually negligible thanks to its big shunt resistance $R_{\mathrm{PD}}$ and low dark current $I_{\mathrm{PD},\mathrm{dk}}^{}$ [36]. The other term is from the TIA circuit including thermal noise $4KT/R_{r}$, input noise current $I_{\mathrm{TIA},c}^{}$ and input noise voltage $V_{\mathrm{TIA},v}^{}$ of the operational amplifier. The electrical gain of TIA amplifies quantum fluctuations as well as the electronic fluctuations, so the electronic noise included in the homodyne raw measurements comes mainly from the amplified TIA circuit noise. LO effectively acts as a noise-less amplifier for the quantum fluctuations of the vacuum state and the electrical noise is independent of the LO. In fact, the optical fluctuations seen by the detector can be made much larger than the electronic fluctuations by increasing the laser intensity of LO beam to enhance the *QCNR* signally [37].

At the same time, the gain of a typical op-amp is inversely proportional to frequency and characterized by its gain–bandwidth product (GBWP). As a trade-off, lower electrical gain put up with higher op-amp bandwidth. In fact, theoretically, vacuum quadrature fluctuates with unlimited bandwidth in the frequency domain. The random number generation rate in this scheme is ultimately limited by the bandwidth of the homodyne detector. Increased bandwidth of op-amp allows higher sampling rate.

## 3. Experiment and Results

Experimentally, we dedicate to enhance quantum entropy in quantum RNG by enhancing the laser power of LO beam to noise-independently amplify quadrature fluctuation of vacuum state on the premise of optimizing ADC sampling range. An extraction ratio of true randomness of 85.3% is achieved by finite enhancement of the LO power when classical noise excursions of the raw data is obvious and the extracted random sequences passed the NIST (National Institute of Standards and Technology), Diehard and the TestU01 tests.

**Figure 3:**
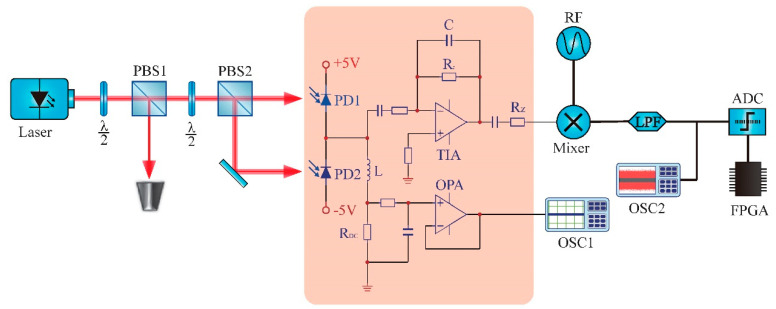
Schematic of the experiment for the quantum random number generator based on homodyne measurements of the quadrature amplitudes of the vacuum state.

The experimental setup is depicted in Figure 3. A 1550 nm laser diode (LD) is driven by constant current with thermoelectric temperature control with a maximal out power of 15 mW. A half-wave plate and a polarizing beamsplitter (PBS2) were combined to serve as accurate 50/50 beamsplitting. Single-mode continuous-wave laser beam from the laser incident into one port of the beamsplitter and acts as the LO, while the other port was blocked to ensure that only the vacuum state could enter in. The vacuum field and the LO interfere on the symmetric beamsplitter to form two output beams with balanced power. The outputs are simultaneously detected by balanced homodyne detector (PDB480C, Thorlabs Inc., Newton, MA, USA) to cancel the excess noise in LO while amplify the quadrature amplitude of the vacuum state, which fluctuates randomly and is independent of any external physical quantities.

**Figure 4:**
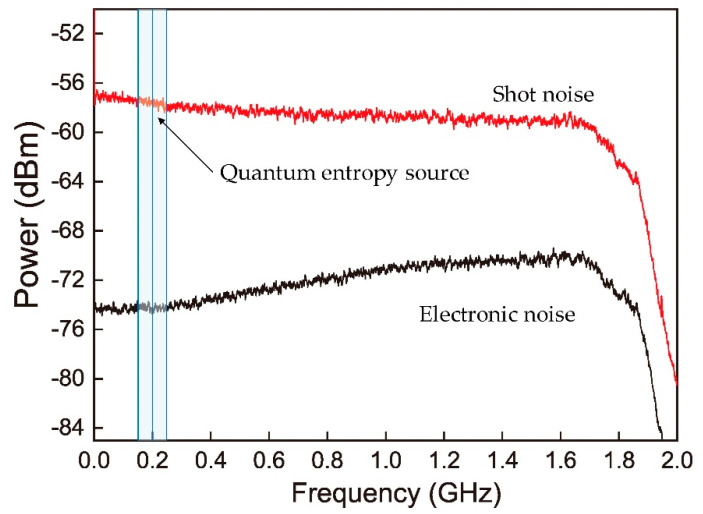
Amplified vacuum noise power spectral when local oscillator (LO) power is 6 mW. 100 MHz vacuum sideband centered at 200 MHz is filtered out as the entropy source of quantum RNG.

Classical noise in the photocurrents is rejected effectively over the whole detection band while the clearance has dependence on frequency as shown in Figure 4. We filtered out a part of the vacuum spectrum, where the clearance is almost consistent, to extract true randomness based on a certain quantum conditional min-entropy and analyze the effect of LO intensity on the conditional min-entropy. The shot noise limited signal from the homodyne detector is mixed down with a 200 MHz carrier (HP8648A) and then passes through a low-pass-filter (LPF) with 50 MHz cut-off frequency (BLP50+, Mini-Circuits Corp., Brooklyn, NY, USA), that is, we actually use 100 MHz vacuum sideband frequency spectrum centered at 200 MHz to act as the random noise resource.

In OHT, BHD system is established and locked to every relative phase to measure the marginal distributions of electromagnetic field quadrature for completely reconstruction of quantum states [25]. While the random numbers generation scheme discussed here focus on a marginal distribution of vacuum state in any one phase thanks to the space rotational invariance of its distributions in the phase space, that is no active modulation or phase (or polarization) stabilization is required.

**Figure 5:**
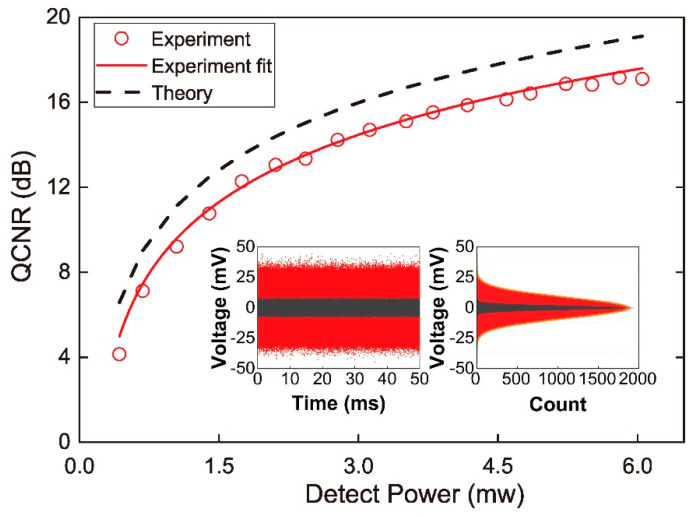
*QCNR* as a function of the LO power. Inset: Resulting histograms of the vacuum (red) and electronic (black) noise obtained at a LO power of 6 mW.

We present the *QCNR* as a function of the LO power arriving at the PD. The electrical noise variance is relatively consistent for certain TIA gain. The clearance depends only on the LO power. The noise power is given by

(15) $$P_{\mathrm{dBm}}=10\mathrm{lg}(\frac{4e^{2}(P/h\nu )\eta BR^{2}}{Z\times 1\;\mathrm{mW}}),$$

where e is the electron charge, η=0.9 is the quantum efficiency of the photodiode (Hamamatsu G8376), B=100 KHz the resolution bandwidth, $R=16\times 10^{3}\;\mathrm{V/A}$ the transimpedance gain of the photo detector and Z=50 Ω the load impedance [38]. For each power value the distribution of the random data was analyzed in time domain in the form of histogram to calculate the *QCNR*. *QCNR* as a function of the LO power figured out from the measured clearance levels is plotted with open circles in Figure 5. The LO power received by each PD is gradually increased from 300 μW to 6 mW by rotating the HWP before PBS1. Here we interpolate between the experimental points to obtain the dependence of *QCNR* on LO power. It is shown as the black dashed line in Figure 5. The experimental results are given by red open circles and can be fitted well by the theoretical curve with a transimpedance gain of $13.1\times 10^{3}\;\mathrm{V/A}$. The experimental results are about 2 dB lower than the theoretically excepted *QCNR*, which is due to uncertainties in determining the transimpedance of the detector and the transmission losses in the LPF.

We increase the LO power up to 6 mW to achieve the largest *QCNR* of 17.8 dB in our system, limited by the maximal output power of the laser. The signal is sampled with a rate of 100 MHz, upper limit of twice the LPF band for the sampling rate to avoid temporal correlation between samples. The resolution is 12 bits and the dynamical range is optimized according to the histogram of the time series acquired with reasonably larger sampling range. The amplitude acquisition scale of oscilloscope (SDA806Zi-A, LeCroy, New York, NY, USA) is continuously adjustable. By choosing the analog-digital conversion range appropriately and tuning the LO intensity finely, the amount of off-scale points can be controlled within allowed statistical deviation. The number of saturated points is easy to restrain on-line from the oscilloscope. The distributions of the random data in time domain and in histogram are shown as insets of Figure 5. The measured total variance of the raw data and electrical noise variance are $154.43\;{\mathrm{mV}}^{2}$ and $5.89\;{\mathrm{mV}}^{2}$, respectively. The classical noise excursions of the raw data are about 17.2 times of the classical noise standard deviation $\sigma_{cl}$. Then the probability distribution of the quantum signal is derived and the conditional min-entropy in the quantum signal is worked out to be 10.13 bit per sample, as circled in red in Figure 2.

**Figure 6:**
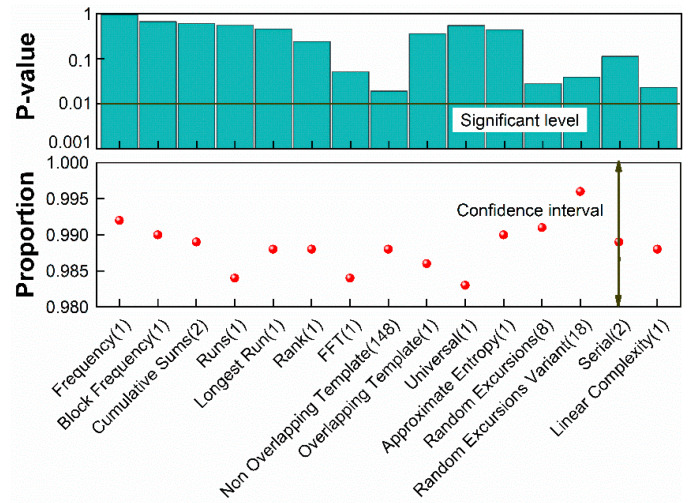
Results of the NIST statistical test suite for a 10^9^-bit sequence.

Finally, information-theoretically provable post-processing scheme, Toeplitz-hashing extractor, is constructed on an FPGA to extract true randomness from the raw data and uniform the Gaussian biased binary stream [39]. A binary Toeplitz matrix of m×n is constructed with a seed of m+n−1 random bits (the seed can be reused since the Toeplitz-hashing extractor is a strong extractor). *m* final random bits are extracted by multiplying the matrix and *n* raw bits, where m/n≤P and $P=H_{\min}(X)/n$. We employ 4096×3520 Toeplitz Hash extractor to distil 10.13 bits/sample. The extraction ratio of 85.3% is the highest as ever reported. We recorded the data with the size of 1 G bits to undergo random test. 1000 sequences with each one 1 M bits are applied to the NIST test and significant level is set as α=0.01. The NIST test is successful if final P-values of all sequences are larger than α with a proportion within the range of $(1-\alpha )\pm 3\sqrt{(1-\alpha )\alpha /n}=0.99\pm 0.00944$ for 15 test suits [40]. P-value shown in the Figure 6 are the worst cases of our test outcomes.

**Figure 7:**
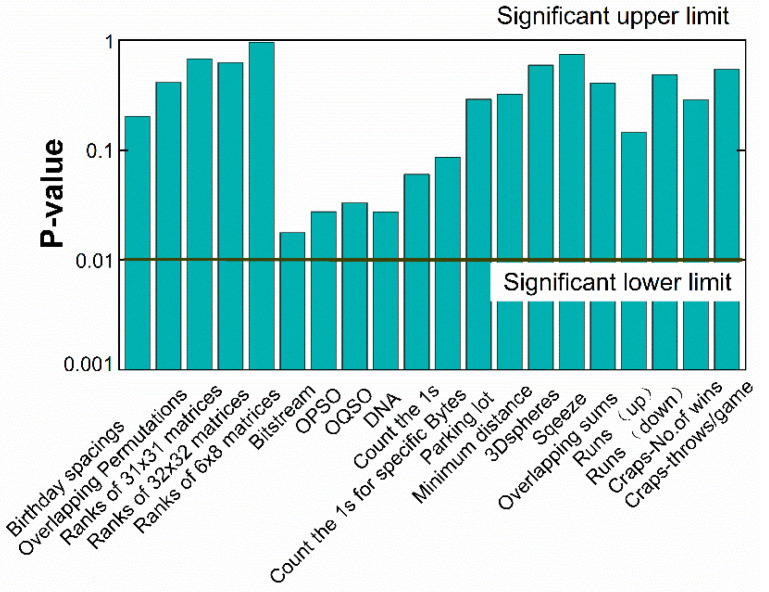
Results of the Diehard statistical test suite for a 10^9^-bit sequence.

Results of the Diehard statistical test suite for the same data file is shown in Figure 7. Kolmogorov-Smirnov (KS) test is used to obtain a final *p*-value to measure the uniformity of the multiple *p*-values. The test is considered successful if all the final *p*-values lies in the range from 0.01 to 0.99 [41].

**Figure 8:**
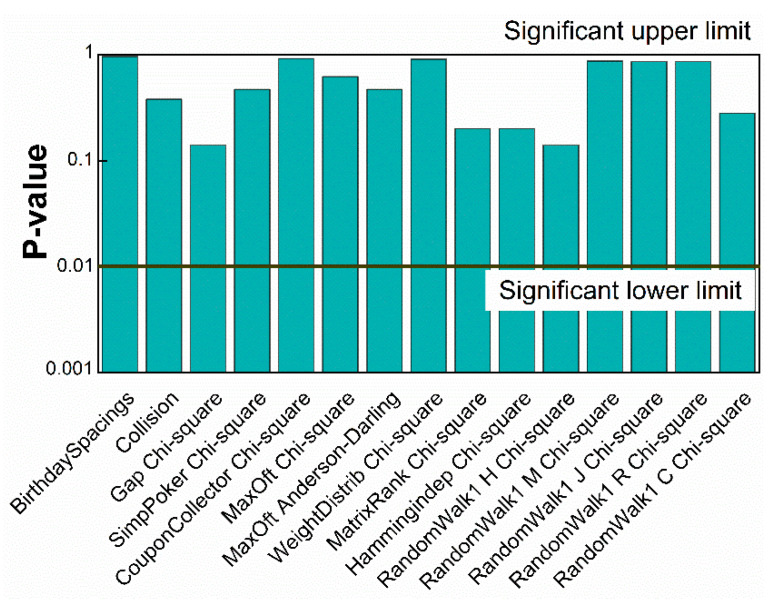
Results of the TestU01 statistical test suite for a 5 × 10^9^-bit sequence.

Constrained by the computational power of crush of TestU01, small crush test is performed with a data size of 8 G bits [42]. The random numbers can pass all the statistical tests successfully. The *p*-value from a failing test converges to 0 or 1. Where the test has multiple *p*-values, the worst case is tabled in Figure 8. All the test items are passed successfully.

On the other hand, we reduce the LO power in the homodyne system to 400 μW and correspondingly, the clearance declines to 4.06 dB. The time series of the system outcomes are collected and statistically analyzed. Classical noise excursion in the Gaussian distribution is about 19.3 times of the classical noise standard deviation. Based on theoretical calculation, the min-entropy is worked out to be 7.73 bits/sample. The hash extraction results with maximum extraction ratio of 0.63 can pass the NIST, Diehard and TestU01 tests finally. 

## 4. Conclusions

To summary, in this work, we discussed the role of LO power plays in random number generation based on quantum detection of vacuum state. When classical noise excursion in the system is trivial, LO power in the homodyne system affect the quantum entropy in raw data insignificantly. Nevertheless, in realistic scenario, the mean of the measured signal distribution is normally nonzero, even much larger noise excursion may be induced deliberately by the eavesdropper. In this case, enough real randomness is attainable only when *QCNR* is high enough. With the LO power enhanced, the vacuum quadrature fluctuations are amplified independent of the electrical noise and the quantum entropy content in the raw data is enhanced effectively. Thus, we propose large dynamical range and moderate TIA gain to pursue higher LO amplification of vacuum quadrature and larger detection bandwidth in homodyne system for higher sampling rate in random numbers generation. Higher hash extraction ratio along with higher sampling rate will enhance the real random number generation rate effectively. More importantly, the quantum RNG system is more robust against to the third part attack.

## 5. Discussion

The central mathematical concept in true RNG is entropy, which is the assessment standard of the security and quality of a RNG. There are many types of entropy. In recent years, min-entropy, a very conservative evaluation, is applied to lower bound the entropy content in quantum RNG and as the indicator for extraction ratio of universal hash extractor. In our work and some ever works [15,19], quantum conditional min-entropy are deduced to impose stricter removal of side signal. Min-entropy is estimated by using the most common value estimate. However, the most common value estimate is more appropriate for IID (independent identically distribution). For non-IID distribution, the estimate may provide an overestimation. The NIST Special Publication 800-90 series of Recommendations provides guidance on the construction and validation of random bit generators (RBGs) in the form of deterministic random bit generators, in which pseudorandom bits are generated by using an unknown seed, or in the form of non-deterministic random bit generators that can be used for cryptographic applications. Entropy source validation is necessary in order to obtain assurance that all relevant requirements of this Recommendation are met. 

**Figure 9:**
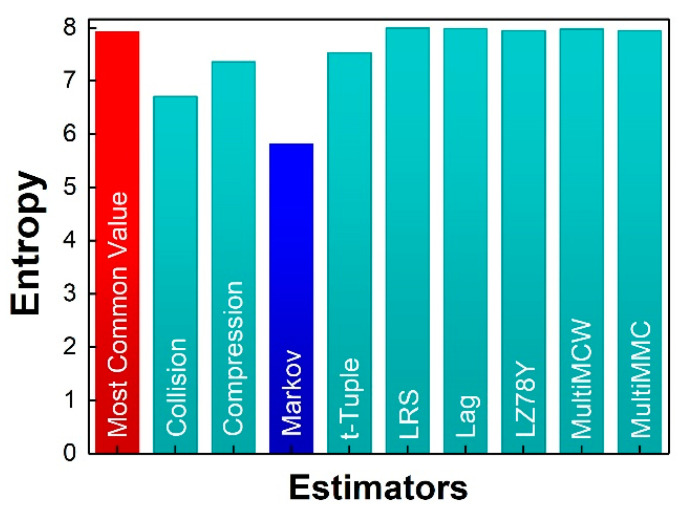
Entropy estimates NIST 800-90B for a 1.6 × 10^6^-bit sequence.

As discussed above, the raw noise-source output in our proposal is biased, Toeplitz hash extractor (conditioning component) is used in the design to reduce that bias to an appropriately level before the RNG exports any bits. For non-IID data, a list of estimators is proposed and the minimum of all the estimates is taken as the entropy assessment of the entropy source for the entropy source validation for the Recommendation. We apply our raw bit strings to the test suit on line [43]. The Test result is shown in Figure 9. Because the size of the sample space in our work is 2^12^, we take the lower 8 bits to meet the applicability of the test. The resulting min-entropy is taken from the minimum of all the estimates as 5.818 per 8 bits. The restart tests are passed. Although the ratio of 72.7% is lower than the evaluation of quantum conditional min-entropy, the quality of our entropy source is validated.
